# The protective effects of systemic dexamethasone on sensory epithelial damage and hearing loss in targeted Cx26-null mice

**DOI:** 10.1038/s41419-022-04987-3

**Published:** 2022-06-10

**Authors:** Kai Xu, Sen Chen, Le Xie, Yue Qiu, Xiao-zhou Liu, Xue Bai, Yuan Jin, Xiao-hui Wang, Yu Sun

**Affiliations:** 1 Department of Otorhinolaryngology, Union Hospital, Tongji Medical College, Huazhong University of Science and Technology, Wuhan, 430022 China; 2 Department of Otolaryngology, Head and Neck Surgery, The Second Affiliated Hospital of Nanchang University, Nanchang, China

**Keywords:** Neurological disorders, Cell death

## Abstract

Mutations in the *GJB2* gene (encoding Connexin26(Cx26)) are the most common cause of hereditary deafness, accounting for about a quarter of all cases. Sensory epithelial damage is considered to be one of the main causes of deafness caused by *GJB2* gene mutation. Dexamethasone (DEX) is widely used in the treatment of a variety of inner ear diseases including sudden sensorineural hearing loss (SSNHL), noise-induced hearing loss (NIHL), and deafness caused by ototoxic drugs. Whether DEX has a direct therapeutic effect on hereditary deafness, especially *GJB2*-related deafness, remains unclear. In this study, we revealed that DEX can effectively prevent hair cell death caused by oxidative stress in cochlear explants. Additionally, two distinct Cx26-null mouse models were established to investigate whether systemic administration of DEX alleviate the cochlear sensory epithelial injury or deafness in these models. In a specific longitudinally Cx26-null model that does not cause deafness, systemic administration of DEX prevents the degeneration of outer hair cells (OHCs) induced by Cx26 knockout. Similarly, in a targeted-Deiter’s cells (DCs) Cx26-null mouse model that causes deafness, treatment with DEX can almost completely prevent OHCs loss and alleviates auditory threshold shifts at some frequencies. Additionally, we observed that DEX inhibited the recruitment of CD45-positive cells in the targeted-DCs Cx26-null mice. Taken together, our results suggest that the protective effect of dexamethasone on cochlear sensory epithelial damage and partially rescue auditory function may be related to the regulation of inner ear immune response in Cx26 deficiency mouse models.

## Introduction

Hereditary hearing loss is the most common hereditary disease that affects every 1.4/1000 newborns worldwide [1]. With the progress of clinical studies, some hereditary diseases, such as hemophilia or retinitis pigmentosa, can be treated well with medication or gene therapy [2–4]. However, there are no effective drugs and successful gene therapy methods for hereditary deafness in clinical practice at present. Cochlear implant, an artificial hearing device, is still not effective for hereditary deafness caused by certain genetic background [5]. Therefore, further exploration of effective drugs remains one potential way to rescue hereditary deafness.

Mutation in the *GJB2* gene is the most common cause of hereditary deafness, accounting for about a quarter of hereditary deafness in different populations [6, 7]. Connexin 26 (Cx26) is widely expressed in the mammals’ inner ear, and it assembles with connexin 30 to form gap junctions (GJs) in adjacent supporting cells (SCs) or fibrocytes of the cochlea [8, 9]. Gap junctions have a relatively large pore that allows ions, microRNAs, secondary messengers, and metabolites ≤1.5 kDa to pass through, providing a channel for material exchange and information communication between cells [10–12]. Cochlear sensory epithelium includes one row of inner hair cells (IHCs) and three rows of OHCs interdigitated among different types of SCs. DCs are a type of SCs. Three rows of DCs correspond to three OHCs. Previous studies have observed that Cx26 deletion induces rapid degeneration of the cochlear sensory epithelium and developmental arrest of the organ of Corti in mouse models [13, 14]. In Cx26 conditional KO mouse models, rapid degeneration of OHCs and SCs were observed at postnatal (P) day 14-P15 and worsened with age [15]. However, the exact mechanism of hair cell death remains unclear. Our recent study revealed that the macrophage-related immune response is involved in the cochlear epithelial injury of Cx26-null mice [16]. In addition, Fetoni et al. reported that redox imbalance induced by dysregulation of the Nfr2 pathway is involved in the accelerated presbycusis caused by partial loss of Cx26 [17]. In this study, oxidative stress was both observed in the inner ear of young and old Cx26-null mice [17]. Therefore, we propose that antioxidant or anti-inflammatory therapy may be a potential target for the treatment of sensory epithelial injury induced by Cx26 deficiency.

Glucocorticoids (GCs) are widely used for a variety of inner ear diseases, such as SSNHL, NIHL, ototoxic drug-induced hearing loss, and Meniere’s disease [18–23]. Dexamethasone (DEX) is a synthetic long-acting glucocorticoid, which has multiple effects such as immunosuppressive, anti-apoptotic, anti-toxin, and anti-allergic effects [24, 25]. As an important member of the GC family, DEX has a good clinical effect in the treatment of SSNHL and preservation of residual hearing in cochlear implant surgery [26–28]. Moreover, intratympanic administration of DEX has been reported to partially ameliorate hearing loss induced by cisplatin [29]. In animal models, intratympanic or systemic administration of DEX has been reported to protect against noise-induced hearing loss [20, 30]. Studies have shown that DEX enhances mitochondrial enzyme activity to stimulate ATP synthesis and improve oxidative energy metabolism [31]. The accumulation of reactive oxygen species (ROS) leading to apoptosis is involved in a variety of cochlear damage mechanisms [32]. However, there is still a lack of direct evidence that DEX can alleviate the oxidative stress injury to the inner ear. Moreover, limited evidence suggests that DEX may prevent post-cochlear-implant damage and inner ear fibrosis in the treatment of hereditary deafness [33]. In an animal study, it has shown that DEX prevents cochlear implantation-induced fibrosis by attenuating IL-1β and TGF-β1 production [34]. However, Kuthubutheen et al. reported that cochlear implantation-induced fibrosis is not improved when systemic DEX was given before the surgery in guinea pigs [35]. So far, there is no direct evidence that DEX has a therapeutic effect on hereditary deafness. Based on the characteristics of GJB2-related hereditary deafness, it is necessary to explore DEX as a therapeutic agent.

In this study, a cochlear explant model of oxidative damage induced by glucose oxidase (GO) was established to investigate the therapeutic effect of DEX on oxidative damage to the cochlear sensory epithelium. Based on the evidence that dexamethasone has anti-oxidant, anti-inflammatory, and anti-apoptotic effects, we hypothesized that DEX may protect against the cochlear sensory epithelium injury or hearing loss induced by Cx26 deficiency. Two distinct Cx26-null mouse models were established to test our hypothesis. One line was DC-targeted Cx26-null mice, which exhibit rapid hair cell loss and deafness [16]. The other line is a specific longitudinally Cx26-null mouse model, in which the degeneration of the third row of outer hair cells (OHC3) was observed at P18 [36]. We assessed the auditory function of Cx26-null mice with or without systemic DEX treatment. In addition, we compared the therapeutic effects of antioxidants with DEX. Furthermore, the degeneration of OHCs and observation of cochlear immune cells was quantified in these models.

## Materials and methods

### Mouse models

All experiments were approved by the Committee of Tongji Medical College, Huazhong University of Science and Technology. Cx26^f/f^ mice were crossed with Fgfr3iCreERT2 or Lgr5CreER mice to obtain Cx26^f/f^; Fgfr3iCreERT2 and Cx26^f/f^; Lgr5CreER mice. All mice were injected with tamoxifen (TMX, T5648-1G, Sigma-Aldrich) subcutaneously at P0 and P1 (the total dose was 1.5 mg/10 g body weight, once a day for two consecutive days) [13, 36]. In Cx26^f/f^; Lgr5CreER mice, Cx26 of the third row of DCs was successfully knocked out [36]. The Cx26^f/f^; Fgfr3iCreERT2 line was used to establish a targeted DCs and PCs Cx26-null mouse model [16].

All mice were raised in the specific-pathogen-free Experimental Animal Center of Huazhong University of Science and Technology. The animals were housed at 22 ± 1 °C under a standard 12 h light/dark cycle and were allowed free access to water and a regular mouse diet.

### Culture of cochlear explants and drug treatments

The preparation of the tail collagen gel matrix has been described in detail in our previous studies [37]. Briefly, the collagen gel matrix was prepared with 9 parts of rat tail collagen (Type 1-4236, BD Biosciences), 1 part of 10× Basal Medium Eagle (BME) solution (B9638, Sigma-Aldrich), and 1 part of 2% sodium carbonate (P1110, Solarbio). Collagen gel matrix was dropped on the culture dish, and 1.3 mL serum-free medium was added after the gel became solid. Wild-type C57BL/6 mice were sacrificed at P3, and the cochleae were quickly separated from the temporal bone. Afterward, the cochlear explants were carefully isolated and placed on the collagen gel matrix. Then, the culture dish was transferred to an incubator at 37 °C with 5% CO_2_ overnight before each treatment. On the following day, the control group aswas cultured with the fresh medium without any drugs, GO group was exposed to 160 U/L glucose oxidase, GO + dexamethasone group was exposed to the same concentration of GO together with 100 μg/mL dexamethasone, or dexamethasone group was exposed to 100 μg/mL dexamethasone. All cochlear explants were incubated for 24 h and harvested for hair cell counting. Cochlear explants after GO treatment were washed in PBS and incubat with 10 mM DCFH-DA (D6883, Sigma-Aldrich) in serum-free DMEM for 30 min.

### Dexamethasone or N-acetylcysteine (NAC) treatment in vivo

Clinically, systemic dexamethasone has demonstrated conclusive benefits in reversing SSNHL despite considerable number of potential side effects. To test the protective effect of dexamethasone on the targeted-cell Cx26-null models. Mice in the dexamethasone intervention group were subcutaneously injected with dexamethasone every two days for eight consecutive days. Two concentrations of DEX (3 mg/kg and 5 mg/kg) were tested in our preliminary study, based on the literature [38, 39]. The dexamethasone was diluted in sterile saline at 0.5 mg/ml and final dose of 5 mg/kg of body weight were used in this study. Mice in the NAC intervention group were subcutaneously injected with NAC (300 mg/kg) every two days for eight consecutive days in this study, the does based on the literature [40]. Control group or Cx26-null group received the same volume of saline.

### Auditory brainstem response (ABR) measurements

The hearing thresholds of each group (*n* = 5) were measured by ABR at P18. The detailed methods for the ABR test have been described in detail in our previous publications [41]. Briefly, mice were deeply anesthetized and body temperature was maintained with an electric blanket. The recording electrode was inserted under the skin of the skull, and the reference electrode and grounding electrode were placed at the tested ear or the contralateral ear. The hearing threshold was assessed at 4, 8, 16, 24, 32, and 40 kHz. Tone bursts stimuli were generated and responses were recorded by the TDT system (RZ6, Tucker-Davis Tech., Alachua, FL, USA).

### Immunofluorescence

For hair cell and macrophages counting, mice were sacrificed at P18. The cochleae were perfused with 4% paraformaldehyde and decalcification with 10% sodium EDTA solution for 48 h, each stretched cochlear preparation was isolated from the cochlea. Cochlear explants in each group were fixed with 4% paraformaldehyde for 1 h at room temperature. After being rinsed three times with 0.1% Tween-20 in PBS (PBST), the flattened cochlear preparations or cochlear explants were incubated in a blocking solution of 5% Bovine Serum Albumin, followed by incubation with primary antibodies: polyclonal rabbit anti-myosin7a antibodies (25-6790, Proteus Bio-Sciences), polyclonal goat anti-sox2 antibodies (sc-17320, Santa Cruz Biotechnology), polyclonal rabbit anti-Cx26 antibodies (512800, Invitrogen) and goat anti-CD45 polyclonal antibody (AF114, R&D Systems) diluted in PBS overnight at 4 °C. The samples were washed three times with PBST and then incubated with secondary fluorescent antibodies (1:200 dilution, Antgene, China) for 2 h at room temperature. Nuclei and F-actin staining were labeled with DAPI and phalloidin (P5282; Sigma, USA) for 10 min. The samples were visualized under a laser scanning confocal microscope (Nikon, Tokyo, Japan).

### Statistical analyses

In vitro and in vivo experiments, each group contained at least four independent samples. All data are shown as the mean ± SD, Statistical analyses were conducted using GraphPad Prism (Version 8.0, GraphPad Software Inc, USA) and SPSS software (version 19, IBM SPSS Statistics, USA). One-way ANOVA followed by a Dunnett multiple comparisons test was used when there was only one factor. Two-way-ANOVA multiple comparisons test was used when two factors were involved. *P* < 0.05 was considered statistically significant.

## Results

### DEX protects mouse cochlear hair cells against GO induced oxidative damage in cochlear explants

**Fig. 1:**
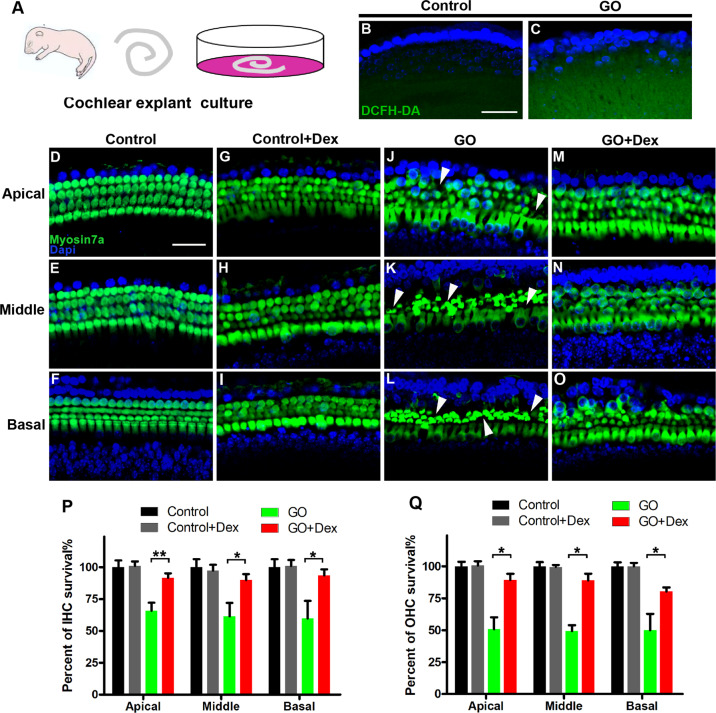
Effects of dexamethasone on glucose oxidase-induced hair cell loss in vitro. **A** Diagram of cochlear explants culture, cochlear basilar membrane dissected from C57BL/6 mice at P3 and cultured in vitro. **B**, **C** The intracellular ROS level was measured with peroxide-sensitive fluorescent probe DCFH-DA in control group and GO group. **D**–**O** Immunofluorescence staining with myosin 7a (green) and DAPI (blue) in different turns of the cochleae from different group. Representative images of the control group, DEX, GO, and GO + DEX group are shown. The white arrowheads in panels **J**–**L** indicate the degeneration of hair cells. **P** Quantification of the survival rate of IHCs in different treatment groups. **Q** Quantification of the survival rate of OHCs in different treatment groups. Data are shown as the mean ± SD, *n* = 5 cochlear explants in each group, **P < 0.01, *P < 0.05. Scale in panels (**B**) and (**D**) represents for 40 μm.

Dysregulation of redox homeostasis is involved in almost all sensorineural hearing loss, such as age-related hearing loss (ARHL), NIHL, ototoxic drug-induced hearing loss, as well as hereditary hearing loss. Recently, DEX was reported to act as a slow-acting free radical cleaner and has been shown to ameliorate hair cell damage induced by various exogenous stresses [42]. In this study, a cochlear explant model of oxidative damage induced by GO was established to explore the therapeutic effect of DEX. Wild-type C57BL/6 mice at P3–P5 were used as the source of cochlear explants (Fig. 1A). Intracellular ROS level was detected by DCFHDA probe, the green fluorescent signal in GO group was much stronger than that of the control group, which indicated that GO treatment significantly increased the intracellular ROS level in the cochlear explant (Fig. 1B, C). After 24 h recovery, explants were cultured in normal medium or with 100 μg/ml DEX only for 24 h. No hair cell degeneration was observed, indicating that DEX does not cause obvious damage to hair cells in cochlear explants (Fig. 1D–I). However, significant loss of hair cells was observed in the GO treatment group (Fig. 1J–L). In contrast, treatment with DEX caused a significant increase in HC survival in different turns, revealing a protective effect by DEX (Fig. 1M–O). Furthermore, we quantified the number of IHCs or OHCs on each turn of the apical, middle, and basal turns in the different groups. In the GO group, quantification (*n* = 5) showed that the survival rate of IHCs were 62.96 ± 5.48, 59.45 ± 9.13, and 57.98 ± 12.0% in the apical, middle, and basal turns, respectively (Fig. 1P). In contrast, the rates of IHC survival in the DEX treatment group were 91.67 ± 3.07, 89.91 ± 4.10, and 93.58 ± 4.10% in the apical, middle, and basal turns, respectively. Similar, the survival rates of OHC in all turns of the DEX treatment group were significantly increased (apical, 53.94 ± 7.32% vs 89.27 ± 4.13%, *P* = 0.0157; middle, 54.32 ± 6.30% vs 88.89 ± 4.62%, *P* = 0.0111; basal, 51.23 ± 11.72% vs 80.25 ± 3.76%, *P* = 0.0421, Fig. 1Q). Our results clearly indicated that treatment with DEX protects HCs from GO-induced oxidative damage.

### The timing of OHC loss was consistent in different Cx26-null mouse models

**Fig. 2:**
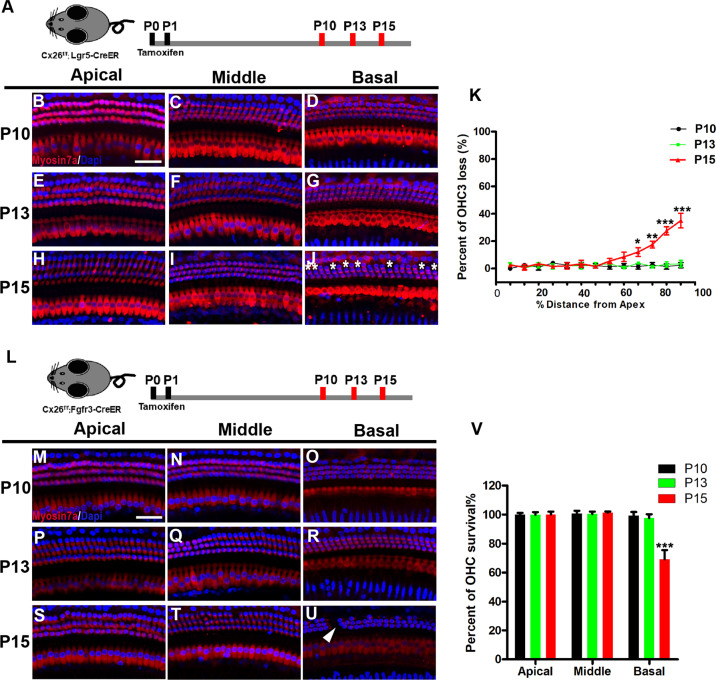
Degeneration pattern of hair cell in Cx26-null mice at different postnatal time points. **A** Schematic of the experimental workflow, Cx26^f/f^; Lgr5-CreER mice were sacrificed at P10, P13, or P15 for examination. **B**–**J** Immunofluorescence staining with myosin 7a (red) and DAPI (blue) in different turns of the Cx26^f/f^; Lgr5CreER mice at different time points, white asterisks indicate the missing OHC3. **K** Quantifications of OHC3 loss at the different time points. **L** Cx26^f/f^; Fgfr3-CreER mice were sacrificed at P10, P13, or P15 for examination. **M**–**U** Immunofluorescence staining with myosin 7a (red) and DAPI (blue) in different turns of the Cx26^f/f^; Fgfr3-CreER mice at different time points, white arrows indicate the missing OHCs in the basal turns of Cx26^f/f^; Fgfr3-CreER mice at P15. **V** Quantifications of OHCs loss at specific cochlear locations at the different time points. *P < 0.05, **P < 0.01, ***P < 0.001. Scale in panels (**B** and **M**) represents for 40 μm.

Our previous study showed that degeneration of OHCs was observed in multiple Cx26-null models at P18. To explore the appropriate treatment window, we dynamically observed hair cell survival in different Cx26-null mouse models (Fig. 2A, L). There was no significant OHC loss observed in the specific longitudinally Cx26-null mice at P10 or P13 (Fig. 2B–G). At P15, a scattered OHC loss was observed in the third row of outer hair cells (OHC3) in the basal turn (Fig. 2H–J). Quantification analysis (*n* = 4) showed that approximately 27.5 ± 2.60% of OHC3 was degenerated in the specific longitudinally Cx26-null group at P15 (Fig. 2K). Additionally, the same time-dependent cell loss pattern was observed in the DC-targeted Cx26-null mice (Fig. 2M–U). The white arrow indicates degeneration of OHCs in the basal turn at P15. Quantitative results show that approximately 30.87 ± 5.45% of OHCs was lost in the DC-targeted Cx26-null mice at P15 (Fig. 2V, *P* = 0.006). Our results indicate that the earliest degeneration of OHCs occurred at P14–P15, suggesting that the window of time for hair cell injury intervention should be earlier than this time point.

### DEX protects against OHC loss better than traditional antioxidantsin specific longitudinally Cx26-null mice

**Fig. 3:**
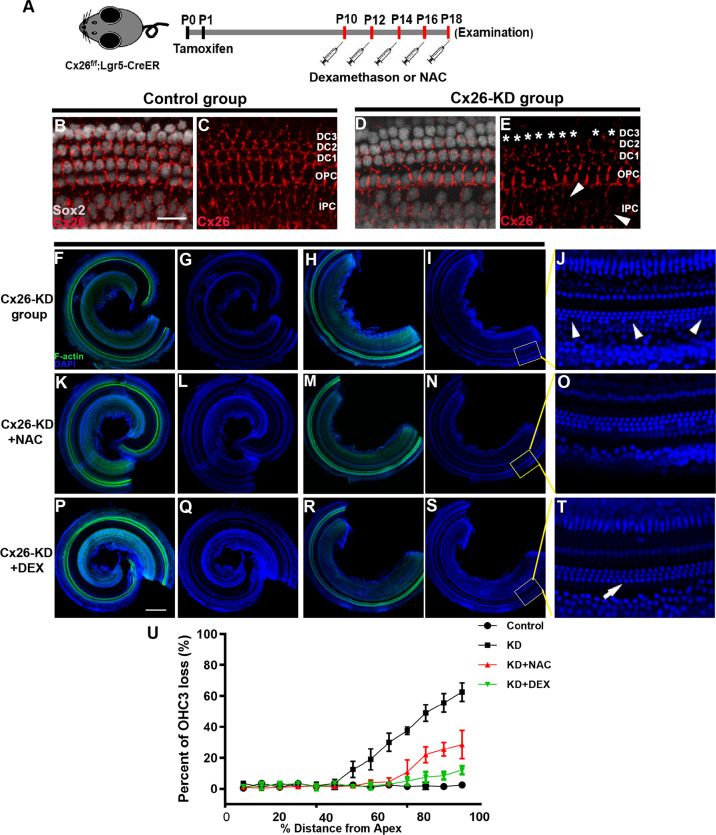
Effects of DEX or NAC on the degeneration of outer hair cell in specific longitudinally Cx26-null mice. **A** Schematic of the experimental workflow, Cx26^f/f^; Lgr5CreER mice were injected with TMX and then treated with dexamethasone 5 mg /kg or NAC 300 mg /kg for five days. **B**, **C** Cx26 immunolabeling (red) in the apical of the control group. **D**, **E** Cx26 immunolabeling (red) in the apical of the specific longitudinally Cx26-null group. The asterisk indicates that Cx26 of the third row DCs was successfully knocked out. **F**–**I** Representative images of the flattened cochlear preparations in specific longitudinally Cx26-null group. **J** Magnifications of the yellow box in the panel (**I**), the white arrowheads in panel J indicated the OHC3 loss. **K**–**N** Representative images of the flattened cochlear preparations in Cx26-null + NAC group. **O** Magnifications of the yellow box in the panel (**N**). **P**–**S** Representative images of the flattened cochlear preparations in Cx26-null + DEX group. **T** Magnifications of the yellow box in the panel (**S**). **U** Quantifications of OHC3 loss in the different groups. Scales in panels (**B**) and (**K**) represent 40 and 200 μm, respectively.

A previous study has shown the presence of oxidative stress in Cx26 knockout models [17], so both DEX and traditional antioxidants (N-Acetylcysteine, NAC) have been used to investigate whether they have protective effects on cochlear sensory epithelial injury caused by Cx26 deficiency. Cx26^f/f^; Lgr5-CreER mice were injected with tamoxifen to establish a specific longitudinally Cx26-null mouse model (Fig. 3A). Because Cx26^f/f^; Lgr5-CreER mice model has less OHC loss and does not show deafness in the early stage [36], this model is more conducive to detecting subtle differences between different drugs. To evaluate the Cx26 knockout pattern in Cx26^f/f^; Lgr5-CreER mice, Cx26 immunofluorescence staining was performed at P7. This showed that nearly all DC3s (asterisks) had lost their Cx26 expression (Fig. 3B–E). Consistent with our previous report, in the specific longitudinally Cx26-null group, scattered OHC3 loss was observed in the basal turn, while the apical and middle turns remained largely intact and showed no substantial OHC loss at P18 (Fig. 3F–J). NAC treatment at 300 mg/kg for five doses can alleviate OHC3 degeneration, however, there is still a scattered OHC3 loss in the Cx26-null+NAC group (Fig. 3K–O). Treating with DEX at 5 mg/kg for five doses almost completely prevents OHC3 loss in the specific longitudinally Cx26-null group (Fig. 3P–T). Furthermore, quantitative analysis (*n* = 5) showed that approximately 49.0 ± 4.58% OHC3 loss was observed in the specific longitudinally Cx26-null group at P18, while the loss of hair cells was significantly reduced in DEX and NAC treatment groups (Fig. 3U). In conclusion, our results demonstrate that treatment with DEX and NAC prevents loss of OHC3s induced by Cx26 deletion in specific longitudinally Cx26-null mice, and the protective effect of DEX was better than that of NAC.

### Treatment with DEX prevents OHC loss and attenuates auditory threshold shifts in DC-targeted Cx26-null mice

**Fig. 4:**
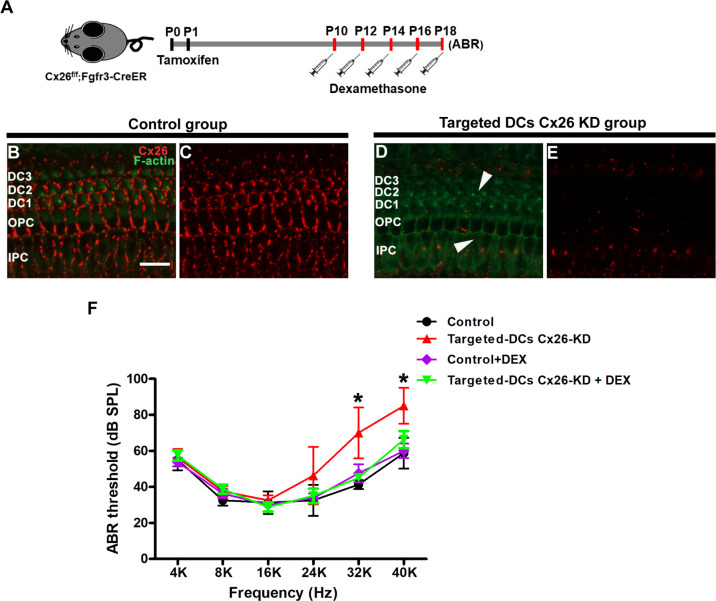
Effects of dexamethasone on the hearing loss in DC-targeted Cx26-null mice. **A** Schematic of the experimental workflow, Cx26^f/f^; Fgfr3-CreER mice were injected with TMX and then treated with dexamethasone 5 mg/kg of body weight for five days. **B**, **C** Cx26 immunolabeling (red) in the apical of the control group. **D**, **E** Cx26 immunolabeling (red) in the apical of the targeted DCs Cx26-null group. The white arrowheads in panel (**D**) indicate that Cx26 of DCs was successfully knocked out. **F** ABR thresholds were analyzed indifferent group at P18. *n* = 5 mice in each group, *P < 0.05. Scale in panel (**B**) represents for 40 μm.

**Fig. 5:**
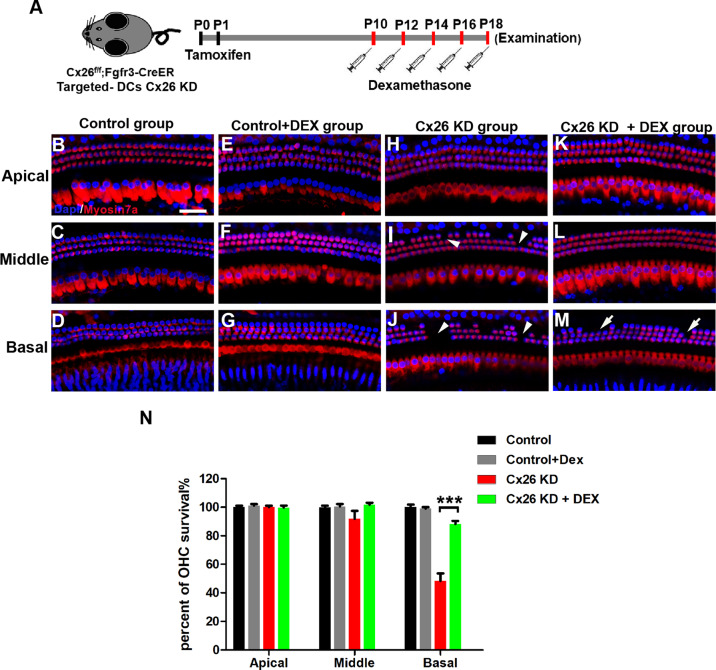
Effects of dexamethasone on the degeneration of outer hair cell in DC-targeted Cx26-null mice. **A** Schematic of the experimental workflow, Cx26^f/f^; Fgfr3-CreER mice were injected with TMX and then treated with dexamethasone 5 mg /kg of body weight for five days. **B**–**M** Immunofluorescence staining with myosin 7a (red) and DAPI (blue) in different turns of the cochleae from different group. Representative images of the control group (**B**–**D**), control + DEX (**E**–**G**), Cx26 -null (**H**–**J**), and Cx26-null + DEX group (**K**–**M**) are shown. The White arrowheads in panels **H**–**J** indicate the miss of hair cells. **N** Quantification of the survival rate of OHCs in different treatment group. *n* = 5 mice in each group, ***P < 0.001. Scale in panel (**B**) represents for 40 μm.

Next, a DC-targeted Cx26-null mouse model was established to explore the protective effects of DEX (Figs. 4A, 5A). In this line, almost all DCs and pillar cells (PCs) were knocked out of Cx26 successfully at P7 (Fig. 4B–E). To determine whether DEX preserves hearing function, the auditory brainstem response (ABR) was evaluated at P18. As shown in Fig. 4F, the hearing thresholds in the control group at 4, 8,16, 24, 32 and 40 kHz were 55.0 ± 5.0, 32.5 ± 2.5, 31.25 ± 5.5, 32.5 ± 7.5, 41.25 ± 2.17 and 58.75 ± 7.40 dB sound pressure level (SPL), respectively. The DC-targeted Cx26-null group showed a high-frequency hearing loss, with the hearing thresholds at 8–40 kHz being 56.25 ± 4.15,37.5 ± 2.5, 32.5 ± 2.5, 46.25 ± 13.86, 70.0 ± 12.25 and 85.0 ± 8.66 dB SPL, respectively. In the DC-targeted Cx26-null group, treatment with DEX significantly attenuated threshold shifts at 32 and 40 kHz with an average reduction of 25 and 18.75 dB, respectively (Fig. 4F, *P* = 0.0305). There is almost no statistical difference in hearing between the Cx26-null+DEX group and the control group at P18. In addition, hair cells were labeled with Myosin7a by immunofluorescence staining. In the control group and the DEX alone treatment group, there was no obvious OHC loss at P18 (Fig. 5B–G). Consistent with our previous report, DC-targeted Cx26 deficiency induced OHC loss in the middle and basal turns (Fig. 5H–J). In line with the reduction of threshold shifts, treatment with DEX also significantly attenuated OHC loss in the middle and basal turns (Fig. 5K–M). Quantification analysis (*n* = 4) showed that the survival rate of OHCs in the basal turn increased from 48.34 ± 4.52 to 88.22 ± 1.91% in the DEX treatment group (Fig. 5N, *P* = 0.0009). These results demonstrate that treatment with DEX prevents OHC loss and alleviates auditory threshold shifts at certain frequencies in the DC-targeted Cx26-null mouse model.

### Treatment with DEX attenuates the recruitment of CD45-positive cells response to hair cell death in the DC-targeted Cx26-null mouse model

**Fig. 6:**
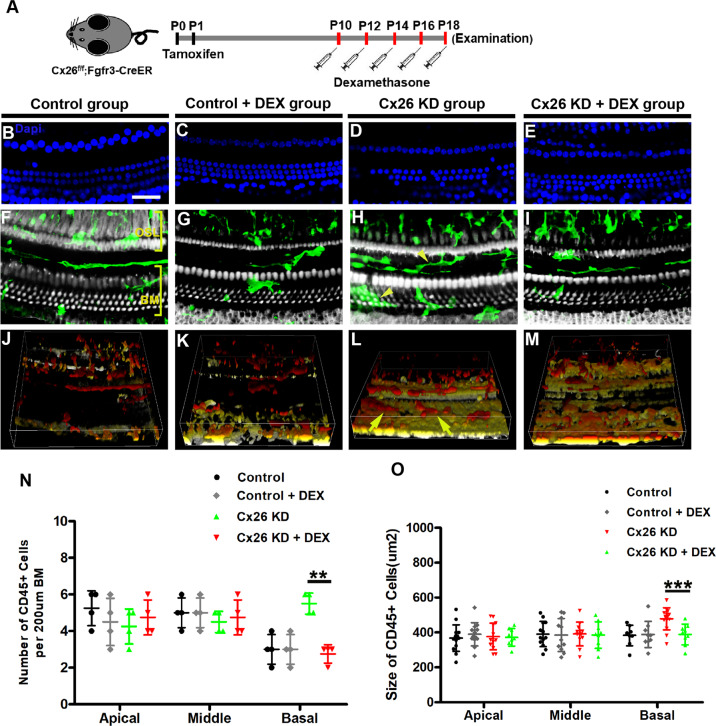
Effects of dexamethasone on the number and morphology of macrophage in DC-targeted Cx26-null mice. **A** Schematic of the experimental workflow, Cx26^f/f^; Fgfr3-CreER mice were treated with TMX and then treated with dexamethasone 5 mg /kg of body weight for five days. **B**–**E** Immunofluorescence staining with DAPI to show the pattern of hair cell damage from different group. **F**–**I** Immunofluorescence staining with CD45 (green) to visualized the distribution and morphology of macrophages in the basilar membrane from different groups. **J**–**M** The three-dimensional reconstruction of CD45 positive cells to show the morphology of CD45 positive cells from different groups. **N** Quantification of the numbers of CD45 positive cells on the scala tympani side of the basilar membrane in different groups. **O** Quantifications of CD45 positive cells size in three turns from different group. *n* = 4 mice in each group, **P < 0.01, ***P < 0.001. Scale in panels (**B**, **C**) represents for 40 μm.

Since our previous study has reported that the macrophage-related immune response is involved in the pathological process of cochlear sensory epithelium damage induced by Cx26 knockout [16]. The number and morphology of macrophage were observed to investigate whether the reduction of hair cell damage by treatment with DEX is associated with inhibition of inner ear immune response. In a previous study, co-staining of F4/80 (another macrophage-specific marker) and CD45 was used to label cochlear immune cells. Most CD45-positive cells with an irregular shape and approximately 80% CD45-positive cells displayed F4/80 immunoreactivity, indicating that these cells are mostly macrophages [43]. Moreover, CD45 was widely used as a cochlear macrophage marker in different studies [44–46]. As shown in Fig. 6, most CD45-positive cells showed irregular appearance and are considered to be basilar membrane macrophages. The number of CD45-positive cells in the basal turn of the DC-targeted Cx26-Null group was significantly increased (Fig. 6H). The DEX treatment group showed dramatically decreased numbers of CD45-positive cells in the basal turn compared with the Cx26-null group (Fig. 6I). Furthermore, quantification of the results showed that the number of CD45-positive cells in the basal turn of the DEX treatment group was reduced significantly in comparison with the Cx26-null group (2.75 ± 0.43 vs 5.5 ± 0.5, *P* = 0.0032, Fig. 6N). The three-dimensional reconstruction of CD45-positive cells to show the morphology of CD45+ cells. In the area of sensory cell damage, CD45-positive cells show enlarged cell bodies as well as amoeboid transformation. We compared the sizes of CD45-positive cells in each group. The average size of CD45-positive cells in the basal turn of the Cx26 KD group was larger than that of the control group (475.37 ± 62.51 mm^2^ vs 382.52 ± 55.29 mm^2^, *P* = 0.00468, Fig. 6O). Meanwhile, after treatment with DEX, the size of CD45+ cells was significantly reduced.

## Discussion

In the clinic, GJB2 mutations cause various auditory phenotypes, including profound sensorineural hearing loss or late-onset progressive hearing loss. This diversity of clinical phenotypes implies that hearing loss induced by Cx26 mutations has various underlying deafness mechanisms. Recent studies have shown that multiple pathological mechanisms are involved in *Gjb2* mutation-related hereditary deafness, in which sensory epithelial damage is considered to be a recognized cause of deafness [47]. In the systemic Cx26 null mouse model, rapid degeneration of OHCs and SCs are observed at P14-P15, and it triggered secondary degeneration of spiral ganglion neurons (SGN) at P30 [48, 49]. An important question is whether the larger the knockout range of Cx26, the earlier the appearance of cochlear cell death. However, when two different Cx26-null models with distinct knockout ranges were established, the timing of cell death in the inner ear is highly consistent. The above results suggest that there is an optimal time window for the intervention of *Gjb2*-related cochlear cell death. Besides, our preliminary study found that mouse pups older than 10 days could tolerate 5 doses of DEX injections, and this total dose of DEX causes death in pups less than 1 week old. It has been reported that deletion of Cx26 contributes to a diminished antioxidant defense system in the cochlea of *Gjb2* knockout mice, indicating that accumulation of ROS might be involved in sensory epithelial damage [17]. Studies from other organ systems also support the interaction between Cx26, oxidative stress, and cell damage [50]. In vitro study, our results indicate that DEX can effectively reduce the oxidative stress injury of HC induced by GO. Therefore, we used a Cx26-null model with very mild OHC3 death and no deafness to verify the effectiveness of DEX and antioxidants [36]. In this line (the specific longitudinally Cx26-null mouse model), DEX treatment protects HCs better than traditional antioxidants. Besides, NAC does not completely block HC death caused by Cx26 deficiency. It suggested that oxidative stress injury may not be the only cause of HC death in the Cx26-null mouse model. In addition to acting as an antioxidant, DEX may block HC death caused by Cx26 deficiency through a variety of pathways.

To further verify the effectiveness of DEX in treating *Gjb2*-related deafness, we established a targeted DC Cx26-null model with both OHC death and deafness. In this line, the location of OHC death was matched with the frequency of hearing loss, suggesting that the hearing loss in this model was mainly caused by OHC death. Our results showed that systemic DEX administration could almost completely prevent OHCs loss and deafness in the short term. So far, how Cx26 knockout of SCs leads to HC death remains unclear. Although we do not know the specific mechanism by which DEX antagonizes OHC death, it indicated that it is a feasible method to save *Gjb2*-related deafness based on protecting OHC death.

There is growing evidence that immune responses are involved in a variety of acute or chronic inner ear diseases including hearing loss caused by acoustic injury, ototoxicity, toxins, and cochlear implantation. In recent years, treatments targeting inflammation have been shown to improve a variety of inner ear diseases. During these cochlear epithelium damage processes, infiltrating or mature tissue macrophages are the main immune response cells, several studies have demonstrated distinct roles for macrophages including antigen presentation, phagocytosis of cellular fragments, and production of inflammatory molecules [46, 51]. Studies have shown that HC damage and deafness induced by noise can be alleviated by regulating the function or the number of macrophages [52, 53]. Sun et al. reported that minocycline inhibition of macrophage activation attenuated neomycin-induced hair cell loss and auditory threshold shifts [54]. In addition, IL-1 blocker therapy significantly improved hearing in patients with Muckle-Wells syndrome, and the hearing improvement effect was related to the time of treatment initiation [55, 56]. Our previous study has shown that immune responses of the inner ear are involved in the cochlear epithelial damage process induced by Cx26 deficiency. However, the expression of cytokines and chemokines in the inner ear of *Gjb2*-related hereditary deafness is significantly different from that of other types of deafness [16, 57]. We still lack direct evidence that cochlear inflammation leads to *Gjb2*-related cochlear sensory epithelial injury and deafness. In this study, we found that treated with DEX significantly inhibited the recruitment of CD45-positive cells in the targeted-DCs Cx26-null mice, suggesting that the protective effect of DEX on OHC injury may be related to the inhibition of inner ear immune response. Our findings provide evidence for the immune response as promising new therapeutic targets for the prevention of *Gjb2*-related hearing loss. More drugs based on inhibition of inflammation in the inner ear could be tried to treat *Gjb2*-related deafness and cochlear sensory epithelial damage.

Up to now, there is still no effective treatment based on the mechanism of *Gjb2*-related hearing loss. Yu et al. inoculated viral vectors into the scala media of conditional Cx26 knockout mice to reconstruct cochlear Cx26 expression, and extensive gap junctions among the supporting cells were successfully reconstructed. Degeneration of HCs and SGNs in conditional Cx26 knockout mice was significantly reduced following exogenous connexin26 expression. Unfortunately, there was no significant improvement in hearing [58]. Our study shows that DEX administration in an optimal time window has a good short-term effect in protecting sensory epithelial cell damage and hearing loss induced by Cx26 deficiency. It suggests that gene therapy combined with glucocorticoids may achieve long-term hearing recovery in the Cx26-null mouse model. Furthermore, whether cochlear implantation combined with glucocorticoid therapy can achieve better clinical efficacy in patients with *GJB2* mutation needs to be further studied.

In conclusion, Cx26-null mice had an optimal deafness treatment window of about one week before the onset of sensory epithelial injury. Oxidative stress injury is an important cause of *Gjb2*-related sensory epithelial death, and antioxidants can partially block HC death. In the targeted-DC Cx26-null model, DEX is very effective in blocking HC death and restoring hearing in the short term. DEX may save *Gjb2*-associated HC death and hearing through a variety of pathways. These new findings have profound implications for the intervention in hereditary deafness caused by Cx26 mutations and show that a more comprehensive understanding of this common hereditary deafness is important and urgent.

## Supplementary information

checklist

## Data Availability

The original data supporting the conclusions of this article will be made available by the authors, further inquiries can be directed to the corresponding author.
